# Sphingosine kinase 1 mediates AGEs-induced fibronectin upregulation in diabetic nephropathy

**DOI:** 10.18632/oncotarget.20205

**Published:** 2017-08-10

**Authors:** Cheng Chen, Wenyan Gong, Changzheng Li, Fengxiao Xiong, Shaogui Wang, Junying Huang, Yu Wang, Zhiquan Chen, Qiuhong Chen, Peiqing Liu, Tian Lan, Heqing Huang

**Affiliations:** ^1^ Laboratory of Pharmacology & Toxicology, School of Pharmaceutical Sciences, Sun Yat-sen University, Guangzhou 510006, China; ^2^ Guangdong Pharmaceutical University, Guangzhou 510006, China; ^3^ National & Local United Engineering Lab of Drug Screening and Evaluation, Guangzhou 510006, China

**Keywords:** AGEs, diabetic nephropathy, sphingosine kinase 1, GMCs, SphK1^-/-^ mice

## Abstract

Activation of sphingosine kinase 1 (SphK1) signaling pathway mediates fibronectin (FN) upregulation in glomerular mesangial cells (GMCs) under high glucose (HG) condition. However, the roles of SphK1 in advanced glycation end products (AGEs)-induced DN have not been elucidated. Here we show that AGEs upregulated FN and SphK1 and SphK1 activity. Inhibition of SphK1 signaling attenuated AGEs-induced FN synthesis in GMCs. Inhibition of AGE receptor (RAGE) signaling reduced the upregulation of FN and SphK1 and SphK1 activity in GMCs induced by AGEs. Treatment of aminoguanidine ameliorates the renal injury and fibrosis in STZ-induced diabetic mice and attenuated SphK1 expression and activity in diabetic mouse kidneys. The renal injury and fibrosis in diabetic SphK1^-/-^ mice was significantly attenuated than WT mice. Furthermore, AGEs upregulated SphK1 by reducing its degradation and prolonging its half-life. Conclusion: SphK1 mediates AGEs-induced FN synthesis in GMCs and diabetic mice under hyperglycemic condition**.**

## INTRODUCTION

Diabetic nephropathy (DN) is a major cause of end-stage renal failure, contributing to the overall morbidity and mortality in diabetic patients [1, 2]. The major pathological characteristics of DN are glomerulosclerosis and tubulointerstitial fibrosis [3]. Under diabetic conditions, proliferation and hypertrophy of glomerular mesangial cells (GMCs) and accumulation of extracellular matrix components such as fibronectin (FN), contribute to the expansion of mesangial area and the thickening of glomerular basement membrane (GBM), leading to renal dysfunction and fibrosis in diabetic patients with nephropathy [4-6]. Augmented non-enzymatic glycosylation of proteins, increased oxidative stress and inflammation under hyperglycemia have been implicated in the pathogenesis of DN [7, 8].

Advanced glycation end products (AGEs) are stable covalent compounds generated from protein, nucleic acid or lipid through nonenzymatic glycation reactions under long-term hyperglycemic condition. AGEs play important roles in the pathological progress of diabetic complications including DN [9-11]. Firstly, AGEs break the interaction between matrix and matrix or matrix and cell, as well as intracellular signaling pathways, and disturbs the physiological structure of kidney in diabetes [12, 13]. Secondly, AGEs cause oxidative stress by inducing the aberrant production of reactive oxygen species (ROS) [14, 15]. Furthermore, by binding to and activating the specific receptor for AGEs (RAGE), AGEs boost intracellular signaling transductions, and mediate the diabetic kidney dysfunction [16]. Our previous study confirmed that AGEs-RAGE upregulated FN and TGF-β1 expression and increased ROS through the Nrf2/ARE anti-oxidative pathway [17]. However, the molecular mechanisms responsible for the induction of DN by AGEs have not been fully understood.

Sphingosine 1-phosphate (S1P) is a bioactive sphingolipid and regulates diverse biological processes, such as cell proliferation, differentiation, and migration [18]. S1P can be secreted out of the cell as a ligand for its specific receptors or stay intracellularly as a second messenger [19]. Sphingosine kinase 1 (SphK1) is the key metabolic enzyme that catalyzes the synthesis of S1P [20]. Recently, the SphK1-S1P signaling pathway has gained increasing attention for its role in the pathogenesis of diabetic renal fibrosis [21, 22]. Hyperglycemia, AGEs, and oxidative stress can activate the SphK1-S1P signaling pathway as well as promote the proliferation of GMCs and the accumulation of fibrotic components [23-25]. We previously demonstrated that activation of the SphK1-S1P signaling pathway increased the FN upregulation and that the function of the SphK1-S1P signaling pathway is closely correlated with AP-1 activation [26, 27]. Moreover, our previous work also suggested that inhibition of SphK1-S1P pathway might delay the progression of DN. However, the effect of further study about deficiency of SphK1 signaling in animal model on the development of DN needs to be illuminated.

Since AGEs and SphK1 are important factors in pathological process of diabetic kidney fibrosis, and SphK1 is also an important downstream protein of AGEs, we propose whether SphK1 signaling activation contributes to the AGEs-induced diabetic kidney fibrosis. Thus, we investigated the roles of SphK1 in AGEs-induced diabetic renal fibrosis and explored the potential mechanism of AGEs-mediated SphK1 signaling. Our current study will provide experimental evidence that SphK1 may be a potential therapeutic target for DN.

## RESULTS

### AGEs induced the expression of FN, SphK1 and SphK1 activity in a dose- and time-dependent manner in GMCs

**Figure 1:**
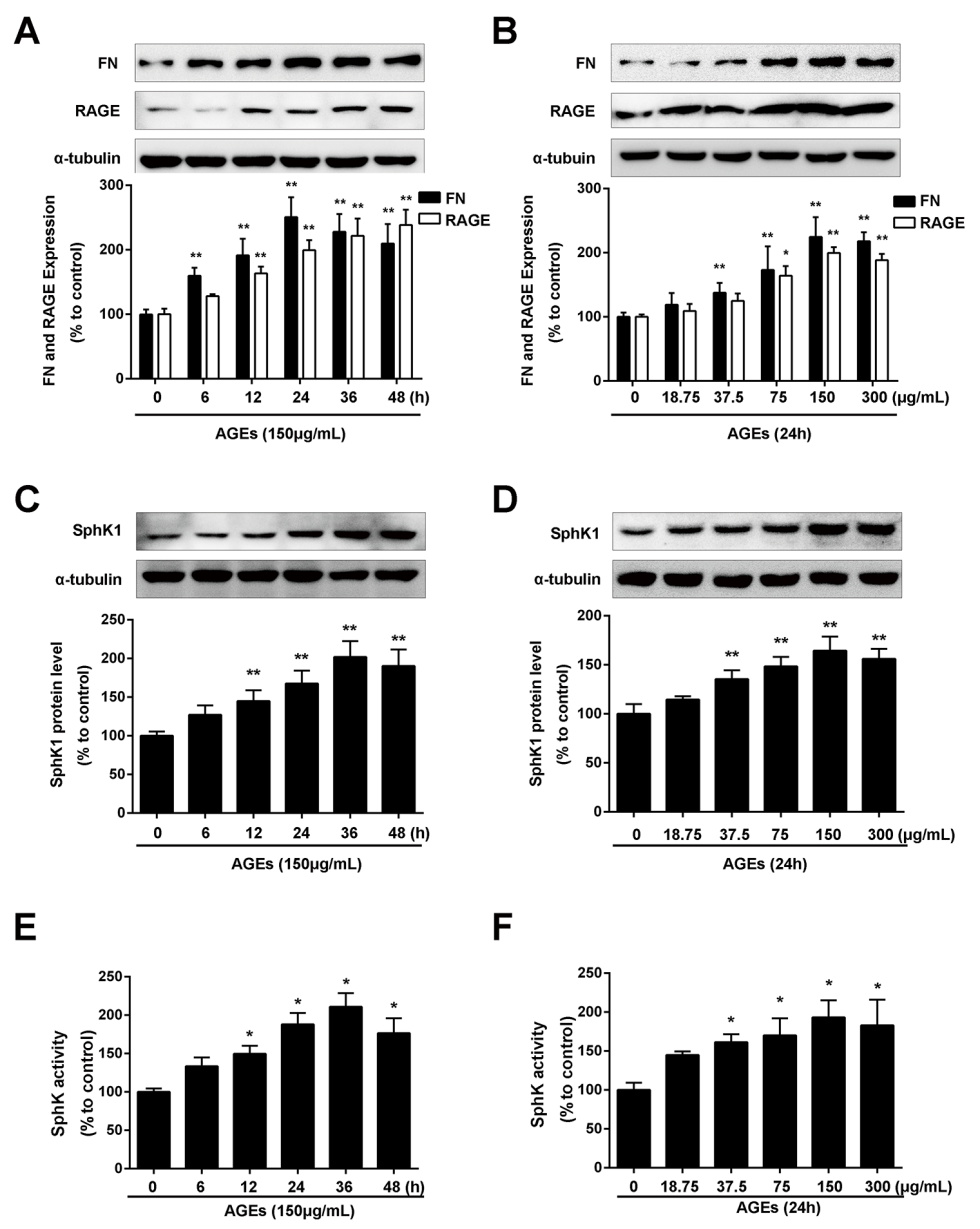
AGEs increased FN and SphK1 protein expression and SphK1 activity in a time- and dose-dependent manner in GMCs The effects of AGEs with different times (150 μg/mL) **(A)** and different concentrations (24 h) **(B)** on the expression of FN and RAGE were determined by western blot assay. AGEs increased the protein expression of SphK1 in a time-**(C)** and dose-**(D)** dependent manner in GMCs. AGEs increased the activity of SphK1 in a time-**(E)** and dose-**(F)** dependent manner in GMCs. * *P* < 0.05, ** *P* < 0.01 vs. 0 μg/mL or 0 h for FN, RAGE and SphK1.

As shown in Figure 1A, AGEs treatment induced the upregulation of FN in a time- dependent manner. The increase in FN expression reached the maximum at 24 h upon AGEs stimulation (*P* < 0.01). AGEs also increased the protein expression of FN in a dose-dependent manner. As shown in Figure 1B, the increase of FN expression reached the maximum in the GMCs exposed to 150 μg/mL AGEs. AGEs also enhanced the expression (Figure 1C and 1D) and activity (Figure 1E and 1F) of SphK1 in a time- and dose- manner along with the upregulation of FN.

### SphK1 mediated the upregulation of FN in GMCs induced by AGEs

**Figure 2:**
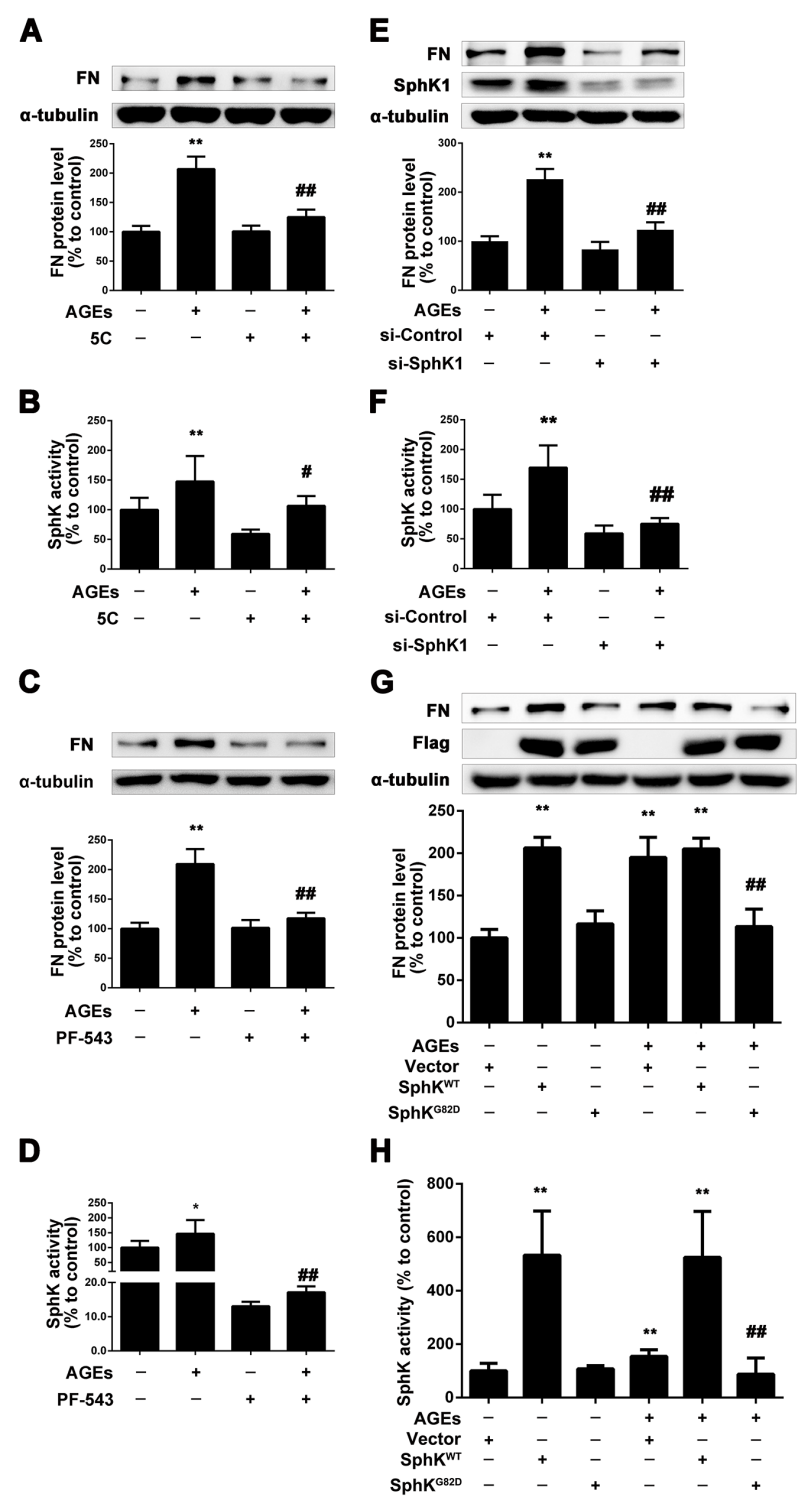
SphK1 mediated the increase of FN in GMCs induced by AGEs 5C attenuated AGEs-induced FN expression and SphK1 activity in GMCs **(A** and **B)**. PF-543 suppressed AGEs-induced FN expression and SphK1 activity in GMCs **(C** and **D)**. SphK1-siRNA decreased AGEs-induced FN expression and SphK1 activity in GMCs **(E** and **F)**. GMCs transfected with empty vector (Vector), wild-type SphK1 (SphKWT) or dominant-negative SphK1 (SphKG82D) were treated with or without AGEs for 48 h **(G** and **H)**. * *P* < 0.05, ** *P* < 0.01 vs. control. # *P* < 0.05, ## *P* < 0.01 vs. AGEs.

To determine whether SphK1 is involved in AGEs-induced overproduction of FN in GMCs, we used 5C and PF-543 [28, 29], the inhibitors of SphK1, and measured the expression of FN in GMCs induced by AGEs. We pretreated the GMCs with 5C (10 μM) or PF-543(1 μM) for 2 h, then we replaced the DMEM and treated the GMCs with 5C (10 μM) or PF-543(1 μM) combined with AGEs (150 μg/mL) for 24 h. As shown in Figure 2A and 2B, 5C reduced the FN expression and SphK1 activity in AGEs-treated GMCs. PF-543 also decreased the expression of FN in AGEs-treated GMCs, and suppressed the activity of SphK1 to 10%, which is more effective than 5C (Figure 2C and 2D).

**Figure 5:**
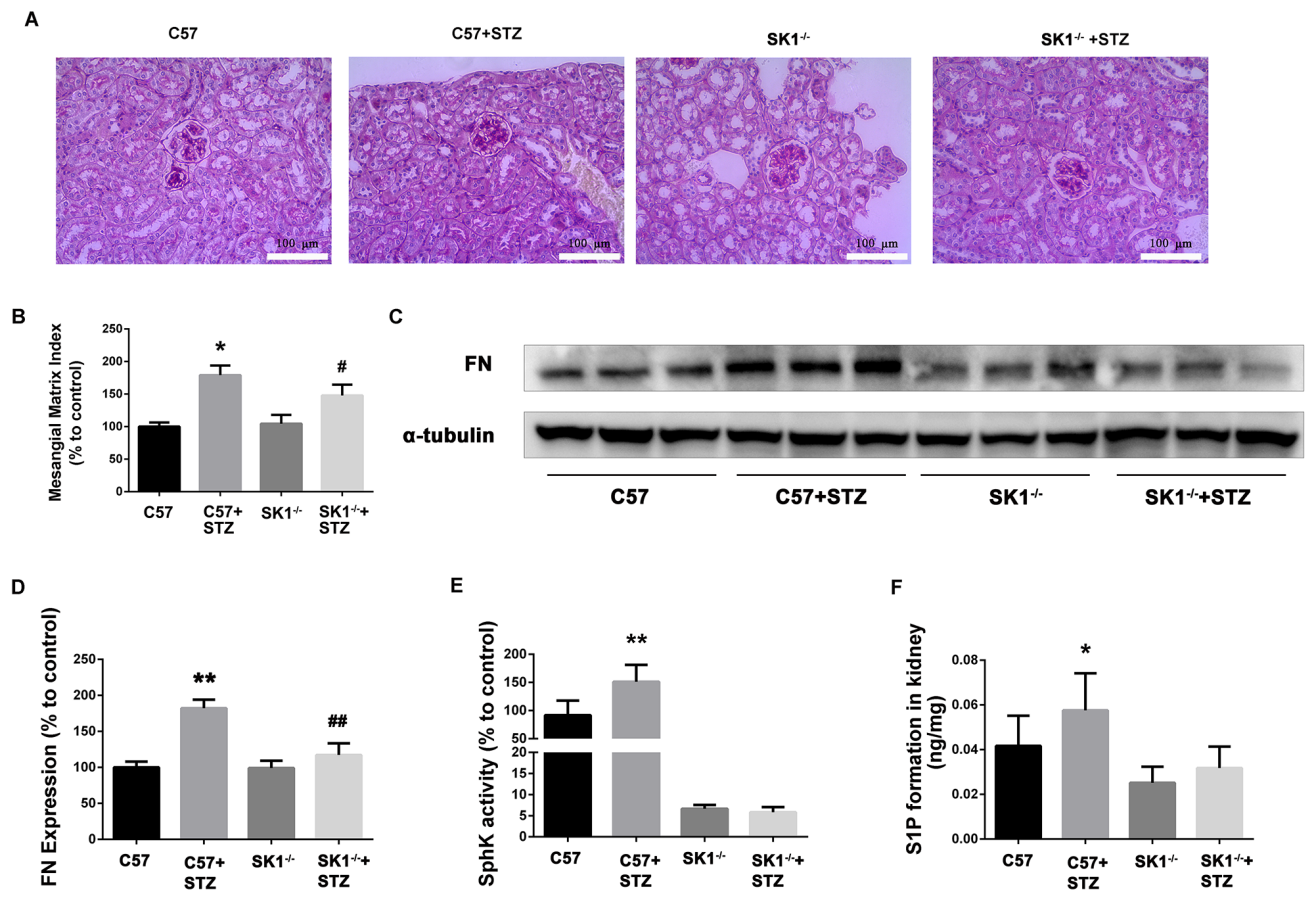
Effects of knock out SphK1 on FN and SphK-S1P signaling pathway in STZ-induced diabetic mice kidney The pathological analysis of glomerular was performed using PAS staining **(A)**. Original magnification, ×400. Mesangial matrix index was defined as the PAS-positive area **(B)**. The expression of FN was evaluated by western blotting **(C)**. The densitometry analysis demonstrated that renal protein levels of FN were increased in untreated diabetic mice and reduced by SphK1 knockout **(D)**. Kidney lysates were subjected to SphK activity assay **(E)**, and Sphingosine 1-phosphate (S1P) quantification **(F)** using LC-MS/MS. *P < 0.05, **P < 0.01 vs. WT, # *P* < 0.05, ## *P* < 0.01 vs. WT+STZ.

To further investigate the role of SphK1 in AGEs-induced GMCs, we selectively silenced SphK1 to observe its effect on FN. Results showed that the expression of FN effectively decreased by SphK1 knockdown treatment for 48 h, which was accompanied with the decreased expression and activity of SphK1 (Figure 2E and 2F).

Next, GMCs were transfected with SphK1 plasmid to investigate whether SphK1-S1P pathway is involved in the AGE-induced FN upregulation. As shown in Figure 2G, overexpression of the wild type plasmid SphK^WT^ could increase the expression of FN in GMCs in the absence or presence of AGEs. However, the dominant-negative SphK1 plasmid SphK^G82D^ attenuated the FN upregulation induced by AGEs in GMCs. The activity of SphK1 in GMCs transfected with plasmid was shown in Figure 2H. These results demonstrated that SphK1 plays an important role in the FN upregulation in AGEs-induced GMCs.

### RAGE mediated the enhanced expression and activity of SphK1 in GMCs induced by AGEs

**Figure 3:**
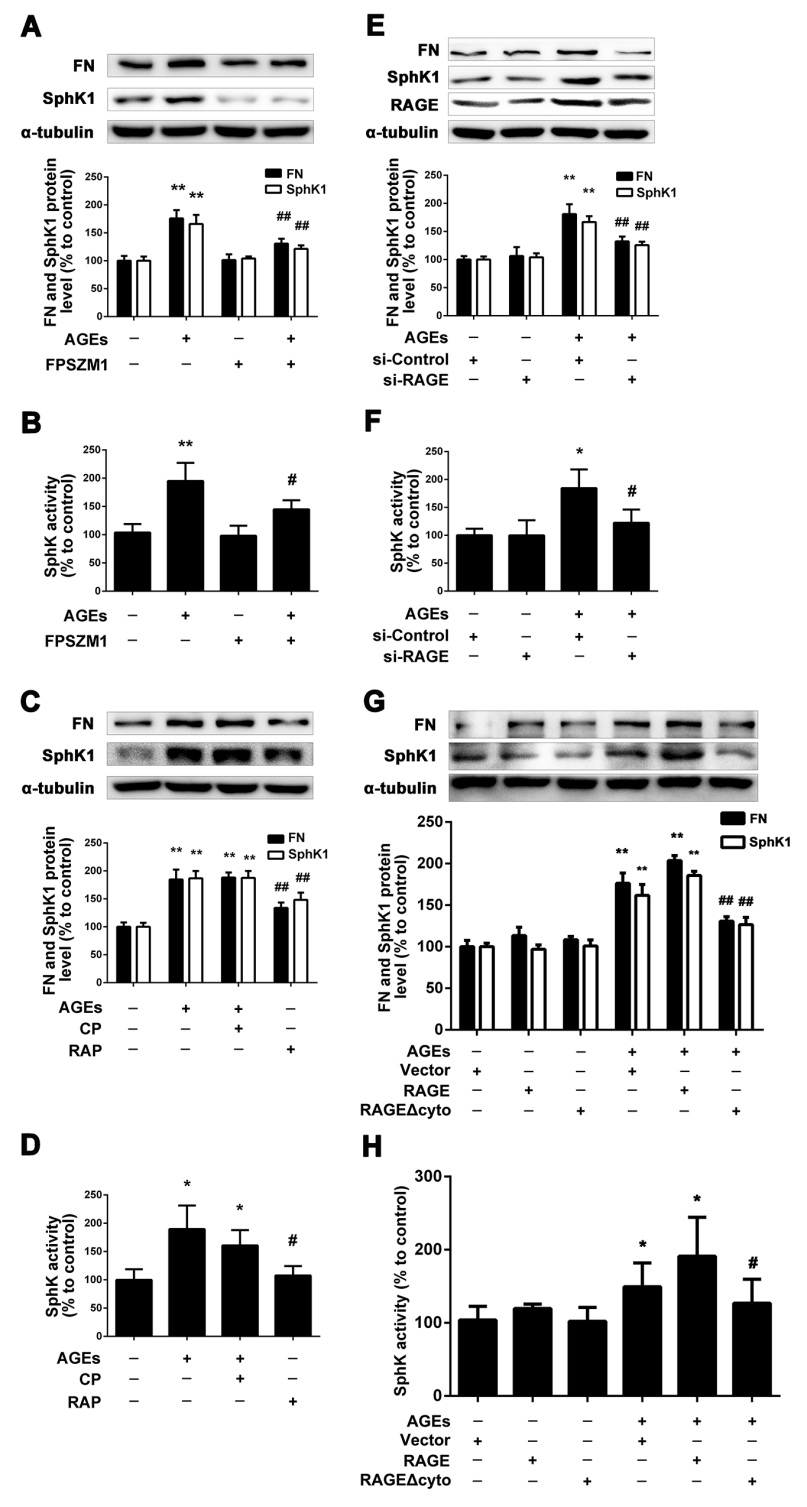
RAGE mediated the regulation of SphK1 upon AGEs treatment FPS-ZM1 attenuated AGEs-induced FN and SphK1 expression and SphK1 activity in GMCs **(A** and **B)**. Blocking of RAGE abolished the effects of AGEs on FN and SphK1 expression and SphK1 activity **(C** and **D)**. RAGE-siRNA decreased AGEs-induced FN expression and SphK1 activity in GMCs **(E** and **F)**. GMCs transfected with empty vector (Vector), RAGE and RAGE Δcyto were treated with or without AGEs for 48 h **(G** and **H)**. * *P* < 0.05, ** *P* < 0.01 vs. control. # *P* < 0.05, ## *P* < 0.01 vs. AGEs.

As SphK1 is involved in AGEs-induced FN expression in GMCs, it raises the questions that how AGEs mediate SphK1 to upregulate FN expression. Firstly, we used FPS-ZM1 [30], a inhibitor of RAGE, to examine the effects of RAGE on the expression of FN and SphK1. We pretreated the GMCs with FPS-ZM1 (1μM) for 1 h, then we replaced the DMEM, and treated the GMCs with FPS-ZM1 (1μM) combined with AGEs (150 μg/mL) for 24 h. As shown in Figure 3A and 3B, FPS-ZM1 markedly reduced the expressions of FN, SphK1 and the activity of SphK1 in AGEs-treated GMCs.

Then, we used RAP [31], a blocking polypeptide, to observe the effect of RAGE on FN and SphK1 by blocking the RAGE and we also used control peptide (CP) as control. We pretreated the GMCs with CP or RAP (10 μM) for 1 h, then we replaced the DMEM and treated the GMCs with CP or RAP (10 μM) and AGEs (150 μg/mL) for 24 h. As shown in Figure 3C and 3D, RAP reduced the expressions of FN, SphK1 and the activity of SphK1 in AGEs-treated GMCs by blocking RAGE, while CP had no effect on the expression of FN and SphK1. Those results further demonstrated that RAGE mediates the process of AGEs-induced the expression and activity of SphK1.

Following that, on the basis of inhibiting and blocking RAGE, we silenced or overexpressed RAGE. As shown in Figure 3E and 3F, the expression of FN was significantly decreased by silencing RAGE for 48 h, accompanied with the decrease in the expression and activity of RAGE. Furthermore, we overexpressed wild-type full-length RAGE or C-terminal cytosolic domain-deleted mutant-type RAGE Δcyto in GMCs. As shown in Figure 3G and 3H, we found that overexpression of wild-type full-length RAGE could further upregulate the expressions of FN and SphK1 as well as the activity of SphK1, while overexpression of RAGE Δcyto could effectively suppress the upregulation of FN and SphK1.

### Inhibition of AGEs formation attenuates the expression and activity of SphK1 in diabetic mouse kidney

For further observation on the effect of AGEs-mediated SphK1, diabetic mice were induced by STZ and treated with aminoguanidine. As shown in Table 1, aminoguanidine effectively reduced the kidney/body weight ratio (KW/BW), blood urea nitrogen (BUN), serum creatinine (Cr) and 24 h albuminuria (24 h UP), compared with diabetic mice. However, aminoguanidine treatment did not reduced fasting blood glucose in diabetic mice. These results suggested that aminoguanidine improved kidney function mainly through inhibition of AGEs formation instead of hypoglycemic effects.

**Table 1:** The effects of AG on the fasting blood glucose, kidney weight/body weight ratio, and renal function parameters of STZ-induced mice

Parameters	CON	STZ	STZ+AG
**Blood glucose(mmol/L)**	5.96±0.43	18.14±3.70	16.03±2.76
**Body weight(g)**	28.19±0.65	24.89±0.75**	28.07±0.97^#^
**KW/BW (%)**	1.22±0.03	1.53±0.14*	1.22±0.05^#^
**Cr(μmol/L)**	14.14±0.57	21.56±2.20*	16.25±1.12^#^
**BUN (mmol/L)**	5.996±0.30	10.04±1.37*	6.491±0.57^#^
**UP 24 h (mg/24 h)**	16.22±0.29	68.50±7.02*	38.88±5.34^#^

Data are represented as Mean ± SD. * *P* < 0.05, ** *P* < 0.01 vs. CON, # *P* < 0.05 vs. STZ.

**Figure 4:**
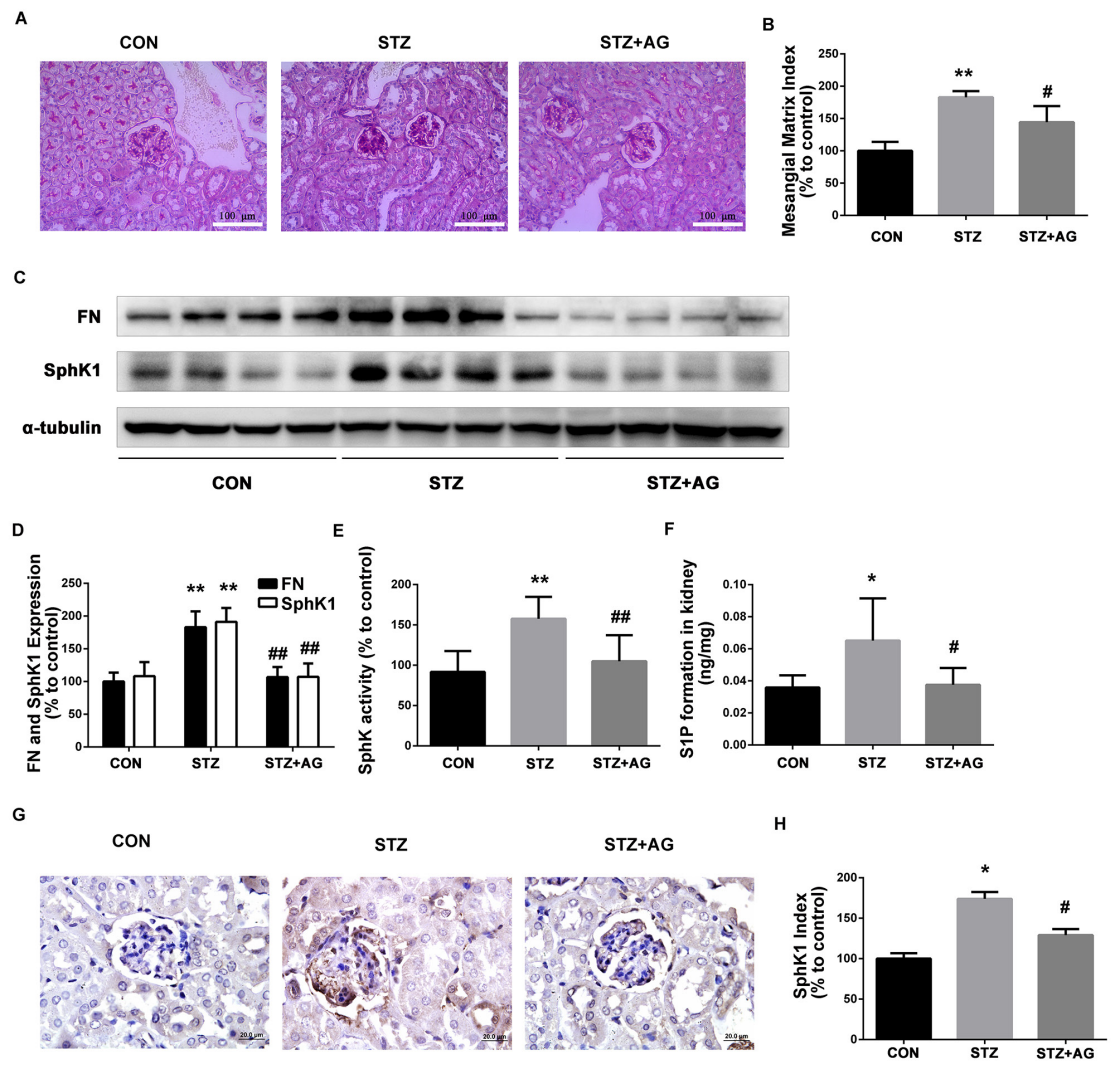
Effects of AG on FN and SphK-S1P signaling pathway in STZ-induced diabetic mice kidney The pathological analysis of glomerular was performed using PAS staining **(A)**. Original magnification, ×400. Mesangial matrix index was defined as the PAS-positive area **(B)**. FN and SphK1 were evaluated by western blotting **(C)**. The densitometry analysis demonstrated that renal protein levels of FN and SphK1 were increased in untreated diabetic mice and reduced by AG treatment **(D)**. Kidney lysates were subjected to SphK activity assay **(E)**, and Sphingosine 1-phosphate (S1P) quantification **(F)** using LC-MS/MS. Immunostaining showed SphK1 (dark brown) expression in glomeruli was increased at 16 weeks after STZ induction and was returned to normal levels by AG treatment **(G)**. The staining of SphK1 was quantified **(H)**. *P < 0.05, ** *P* < 0.01 vs. CON, # *P* < 0.05, ## *P* < 0.01 vs. STZ.

Next, PAS staining showed that aminoguanidine treatment significantly reduced the predominant forms of glomerular alterations in diabetic mice containing increased mesangial matrix areas and regional adhesion of glomerular tuft to the Bowman’s capsule (Figure 4A). To estimate the effect of aminoguanidine treatment on mesangial matrix accumulation, we assessed the mesangial matrix index (MMI). MMI was significantly increased in diabetic mice compared with the control mice (Figure 4B, *P* < 0.05). Aminoguanidine treatment significantly reduced the MMI in the glomeruli of diabetic mice (Figure 4B, *P* < 0.05).

As illustrated in Figure 4C and 4D, FN protein expression obviously increased in the kidneys of diabetic mice (*P* < 0.01). After aminoguanidine treatment, the upregulated FN was significantly inhibited (*P* < 0.01). Collectively, these results confirmed that aminoguanidine could ameliorate renal injury and downregulate the expression of FN in diabetic mouse kidneys.

To determine whether SphK1 was activated in diabetic mouse kidneys, SphK1 tissue distribution and protein expression, activity, and S1P production were evaluated. As shown in Figure 4C and 4D, the expression of SphK1 obviously increased in diabetic mice kidneys (*P* < 0.05). After aminoguanidine treatment, SphK1 protein level was significantly attenuated (*P* < 0.05). Similarly, compared with the control group, SphK activity and S1P levels were increased (Figure 4E and 4F, *P* < 0.05), respectively. After aminoguanidine treatment, SphK activity and S1P formation levels were significantly reduced (Figure 4E and 4F, *P* < 0.05). As shown in Figure 4G and 4H, SphK1 was predominantly expressed in glomeruli assessed by immunohistochemistry. Basic SphK1 staining was detected in the kidneys of normal mice. However, increased staining of SphK1 was observed in the diabetes mouse kidney. By contrast, SphK1 staining markedly decreased in the aminoguanidine-treated mouse kidney. These findings demonstrated that the SphK1-S1P pathway was activated in diabetic mouse kidney and aminoguanidine could attenuate the activation of the SphK1-S1P pathway under hyperglycemic condition.

### SphK1 knockout could improve STZ-induced DN in mice

To further examine the effects of SphK1 on DN, we induced diabetic SphK1 knockout (SphK1^-/-^) mice by STZ. As shown in Table 2, KW/BW, BUN, Cr and 24 h UP levels were increased in wild type (WT) and SphK1^-/-^ diabetic mice (*P* < 0.05). In comparison with diabetic WT mice, SphK1^-/-^ diabetic mice significantly reduced the KW/BW and BUN (*P* < 0.05). Cr and 24 h UP were also declined although there was no significant difference. The results were indicated that SphK1 deficiency improved the STZ-induced DN in mice

**Table 2:** The effects of knock out SphK1 on the fasting blood glucose, kidney weight/body weight ratio, and renal function parameters of STZ-induced mice

Parameters	WT	WT+STZ	SK1^-/-^	SK1^-/-^+STZ
**Blood glucose(mmol/L)**	5.94±0.30	18.18±2.57**	7.3±0.43	16.84±1.73
**Body weight(g)**	27.67±0.65	23.91±0.74*	28.98±0.95	26.03±0.97
**KW/BW (%)**	1.25±0.06	1.59±0.10*	1.40±0.03	1.30±0.07^#^
**Cr(μmol/L)**	20.99±2.37	41.40±5.01*	27.25±6.68	30.39±4.15
**BUN (mmol/L)**	6.381±0.40	12.68±0.47**	7.824±0.59	8.793±0.28^##^
**UP 24 h (mg/24 h)**	20.84±1.63	54.03±5.84**	23.94±2.85	34.34±8.85

Data are represented as Mean ± SD. * *P* < 0.05, ** *P* < 0.01 vs. WT, # *P* < 0.05, ## *P* < 0.01 vs. WT+STZ.

PAS staining showed that the glomerular tuft area was significantly increased in diabetic mice, which was reduced in diabetic SphK1^-/-^ mice (Figure 5A). MMI was obviously increased in diabetic WT mice as compared with the control group. SphK1 deficiency significantly reduced the MMI in the glomeruli of diabetic mice (Figure 5B, *P* < 0.05).

As illustrated in Figure 5C and 5D, FN expression obviously increased in the kidney of diabetic WT mice (*P* < 0.01). However, FN expression was downregulated in SphK1^-/-^ mouse kidney (*P* < 0.01). Consistently, SphK activity and S1P formation were attenuated in SphK1^-/-^ mouse kidney compared with diabetic WT control (Figure 5E and 5F). Collectively, these results confirmed that aminoguanidine could ameliorate renal injury and downregulate the expression of FN in diabetic mouse kidneys.

### SphK1 deficiency ameliorates AGEs-induced DN

**Figure 6:**
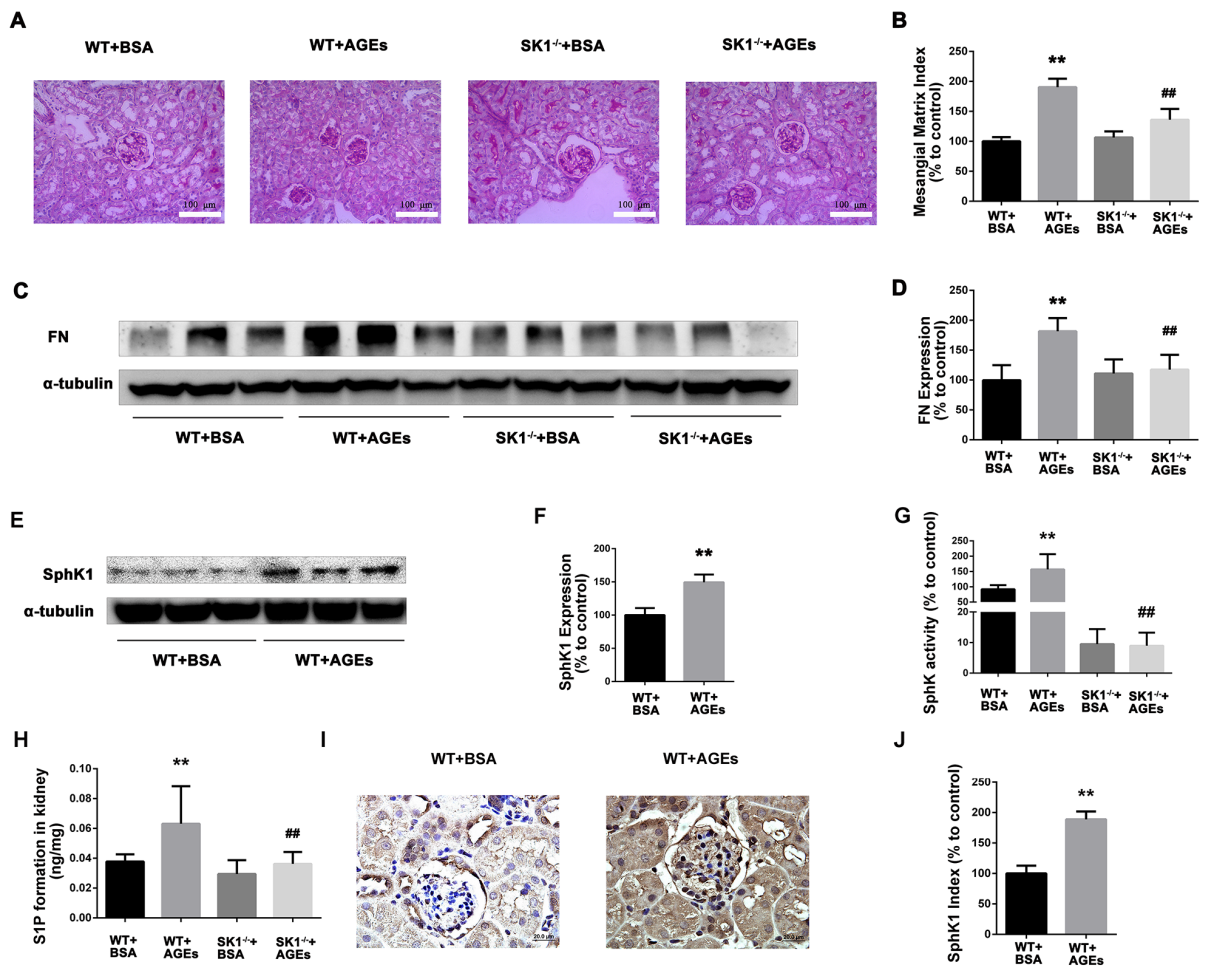
Effects of knock out SphK1 on FN and SphK-S1P signaling pathway in AGEs-induced mice kidney The pathological analysis of glomerular was performed using PAS staining **(A)**. Original magnification, ×400. Mesangial matrix index was defined as the PAS-positive area **(B)**. The expression of FN was evaluated by western blotting **(C)**. The densitometry analysis demonstrated that renal protein levels of FN were increased in untreated diabetic mice and reduced by SphK1 knockout **(D)**. The expression of SphK1 was evaluated by western blotting **(E)**. The densitometry analysis demonstrated that renal protein levels of FN were increased in AGEs-induced mice **(F)**. Kidney lysates were subjected to SphK activity assay **(G)**, and Sphingosine 1-phosphate (S1P) quantification **(H)** using LC-MS/MS. Immunostaining showed SphK1 (dark brown) expression in glomeruli was increased at 12 weeks after AGEs induction **(I)**. The staining of SphK1 was quantified **(J)**. **P < 0.01 vs. WT+BSA, ## *P* < 0.01 vs. WT+AGEs.

For further examine the effects of AGEs and SphK1 on DN, we next used diabetic mice induced by AGEs. As shown in Table 3, BUN and 24 h UP levels were increased in AGEs model mice (*P* < 0.05), indicating the appearance of renal dysfunction. In comparison with the C57 AGEs model mice group, SphK1^-/-^ AGEs model mice effectively reduced the BUN and 24 h UP levels (*P* < 0.05). The results were indicated that SphK1 deficiency attenuated AGEs-induced DN in mice. PAS staining showed that mesangial matrix expansion was attenuated in AGEs-induced SphK1^-/-^ mice (Figure 6A and 6B). Furthermore, FN expression was significantly increased in the kidneys of WT mice, while decreased in SphK1^-/-^ mice (Figure 6C and 6D). Collectively, these results confirmed that knock out SphK1 could ameliorate renal injury and downregulate the expression of FN in AGEs-induced mice kidneys.

**Table 3:** The effects of knock out SphK1 on the kidney weight/body weight ratio, and renal function parameters of AGEs-induced mice

Parameters	WT+BSA	WT+AGEs	SK1^-/-^+BSA	SK1^-/-^+AGEs
**Body weight(g)**	30.69±0.49	29.64±0.46	27.75±0.89	30.18±1.09
**KW/BW (%)**	1.21±0.01	1.18±0.01	1.33±0.03	1.32±0.04
**Cr(μmol/L)**	16.73±1.14	15.59±1.41	18.52±2.73	18.48±1.85
**BUN (mmol/L)**	6.30±0.60	9.47±0.63*	6.90±0.42	6.72±0.71^#^
**UP 24 h (mg/24 h)**	26.53±2.72	39.10±4.84*	23.89±3.24	24.86±3.91^#^

Data are represented as Mean ± SD. * *P* < 0.05, vs. WT+BSA, # *P* < 0.05, vs. WT+AGEs.

Consistently, the expression and activity of SphK1 were significantly increased in AGEs-induced WT mouse kidney, while significantly decreased in AGEs-induced SphK1^-/-^ mice (Figure 6E-6G). Similarly, S1P formation was also decreased in AGEs-induced SphK1^-/-^ mice (Figure 6H, *P* < 0.05). In addition, weak SphK1 staining was detected in the kidneys of normal mice. In contrast, increased expression of SphK1 was observed in the AGEs-induced mice (Figure 6I and 6J). These findings demonstrated that the activation of SphK1-S1P pathway was involved in AGEs-induced DN.

### AGEs inhibit SphK1 degradation through decreasing its ubiquitination

Post-translational modification of proteins is an important way to regulate protein function. Ubiquitination, one of post-translational modification of proteins, could alter protein degradation and protein function. Abnormal ubiquitination has been observed in more and more disease progression [32, 33]. Numerous studies report that SphK1 could be phosphorylation, acetylation and ubiquitination [34-36]. Phosphorylation of SphK1 could increase the activity of SphK1. It remains unclear that whether the ubiquitination of SphK1 is important to regulate SphK1.

**Figure 7:**
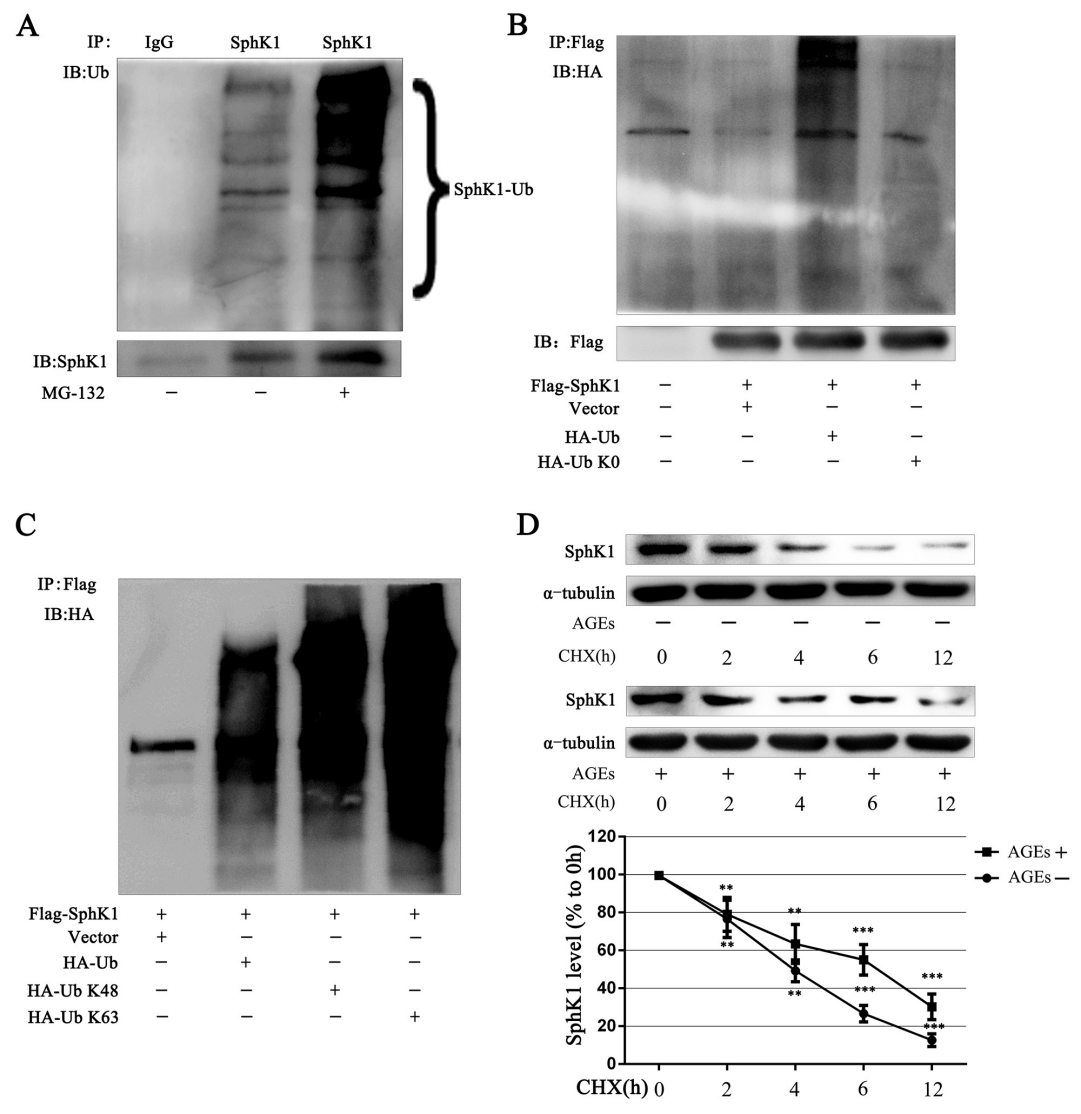
SphK1 could be ubiquitinated and AGEs stabilized the SphK1 protein in GMCs SphK1 was enriched by immunoprecipitation reaction in GMCs and subjected to western blot assay for detecting SphK1 ubiquitination **(A)**. Flag-SphK1 plasmid and pcDNA3-HA-Ub or pcDNA3-HA-Ub K0 plasmid was co-transfected in HEK-293A cells. After 3h of MG-132 treatment, cells were lysed to conduct immunoprecipitation reaction against SphK1 **(B)**. HEK-293A cells were co-transfected with Flag-SphK1 and vector pRK5, pRK5-HA-Ub, pRK5-HA-Ub K48, or pRK5-HA-Ub K63 for 48h. Then Flag immunoprecipitation reaction was performed with anti-Flag antibody and immunoblotted with anti-HA antibody **(C)**. CHX (1 μg/mL) was used to inhibit new protein synthesis in GMCs for detecting SphK1 stabilization **(D)**. ** *P* < 0.01, *** *P* < 0.001 vs. 0 h.

Immunoblots showed that SphK1 protein exhibited a series of high-molecular arginines weight species in GMCs without any treatment, demonstrating that SphK1 can be ubiquitinated. The high-molecular bands of SphK1 were further deeper exposed to the proteasome inhibitor MG-132 (5 μM) for 3 h, demonstrating that SphK1 cannot be degraded when proteasome was inhibited and the ubiquitination of SphK1 protein was increased (Figure 7A).

HA-Ub K0 is a mutant version of HA-Ub that converts all the 7 lysines of ubiquitin intoresulting in the failure to form ubiquitin chains. The co-transfection of the SphK1 plasmid with wild-type HA-Ub or HA-Ub K0 in HEK-293A cells showed that HA-Ub increased exogenous SphK1 ubiquitination and that HA-UbK0 could not ubiquitinate SphK1, indicating the existence of SphK1 polyubiquitination (Figure 7B).

K48-linked ubiquitin chains generally mark substrate proteins for 26S proteasomal-mediated degradation, whereas K63-linked ubiquitin chains mediate various nondegradative functions, including DNA repair, signaling transduction, and protein-protein interactions [37, 38]. Then, we examined what type of ubiquitin chains modified SphK1 protein. We cotransfected Flag-SphK1 with wild-type ubiquitin (HA-Ub), K48 only ubiquitin (HA-Ub K48), or K63 only ubiquitin (HA-Ub K63) and then enriched Flag protein to measure SphK1 ubiquitination in HEK-293A cells. Similar to HA-Ub, both HA-UbK48 and HA-Ub K63 increased SphK1 ubiquitination, as compared with the vector, indicating that K48-linked and K63-linked ubiquitin chains modified SphK1 (Figure 7C).

Because SphK1 could be modified by K48-linked ubiquitin chains, we speculated that AGEs could be regulated SphK1 protein level by ubiquitination of SphK1. We then assessed CHX to inhibit the synthesis of new protein in GMCs and observed the effect of AGEs on SphK1 protein stabilization. As shown in Figure 7D, the half-life of SphK1 was approximately 3 h under normal condition, whereas AGEs significantly up-regulated SphK1 level and prolonged its half-life to 6 h, suggesting that AGEs stabilized SphK1 protein.

## DISCUSSION

AGEs were important factors in pathological process of diabetic kidney fibrosis [16]. Our previous study proved that SphK1 pathway plays a pivotal role in HG-induced FN expression in GMCs, the effect of SphK1 was closely related with the activated transcription factor AP-1 [26, 27]. Here we further observed the regulation of SphK1 in AGEs induced diabetic kidney fibrosis. The vitro study proved that AGEs upregulated the expression of FN and SphK1 as well as the activity of SphK1 in GMCs in time- and dose- dependent manner. SphK1 inhibitors, si-SphK1 or dominant-negative SphK1 plasmid SphK^G82D^ could reverse the addition of FN expression in AGEs-induced GMCs; using RAGE inhibitor, RAGE special blocking polypeptide, si-RAGE, overexpress C-terminal cytosolic domain-deleted mutant-type RAGE Δcyto could reverse the addition of FN and SphK1 expression and the activity of SphK1 in AGEs-induced GMCs. It is preliminary proved that SphK1 plays an important role in upregulating the expression of FN in AGEs-induced GMCs.

Multiple injections of lower doses of STZ was a method that widely used to create animal models of diabetes [39]. Our current data showed that compared with diabetic group, blood glucose was not changed, but kidney function, MMI, the expression of FN were significantly improved in aminoguanidine treatment group. Meanwhile, the expression and activity of SphK1 and S1P formation were markedly reduced in aminoguanidine treatment group. It was indicated that the function of diabetic mouse kidney could be improved without obviously change of blood glucose by attenuation of SphK1 expression and activity after inhibition of AGEs formation.

SphK1 is an important mediator in DN. However, there is a debate whether inhibition SphK1 was beneficial or harmful in DN. Dania found that inhibiting the formation of S1P by knockout SphK1 reduces tubulointerstitial renal inflammation and fibrosis in diabetic nephropathy [40]. Stephanie found that SphK1 expression was elevated in fibroblasts and mesangial cells from SphK2^-/-^ mice compared with wild type mice, and SphK2 deficiency increases proliferation and migration of renal mouse mesangial cells and fibroblasts [41]. Ren found that SphK1 expression increased in the podocytes of kidney sections of patients with diabetic nephropathy and exacerbation of disease was detected by increased albuminuria and CTGF expression in SphK1 deficient mice [42]. The explanation of controversy in the literature may be due to fact that the function of SphK-S1P-S1PR was complexity in different stages of pathological process and different types of cell. Our results were found that blood glucose was elevated in diabetic WT and SphK1 ^-/-^ mice. However, renal function, MMI, FN expression, SphK activity and S1P formation were significantly improved in diabetic SphK1^-/-^ mice compared with diabetic WT mice, suggesting that intervention of SphK1 pathway may has beneficial effects in the treatment of DN.

Because HG could induce multiple changes such as activated polyol signaling pathway, glucolipid metabolism derangements and increased nonenzymatic glycation of proteins, using tail vein injection or intraperitoneal injection AGEs could directly explain the effect of AGEs in DN. Yang CW created model by using intraperitoneal injection of AGEs for four weeks, they found that mRNA of collagen IV and TGF-β1 were increased in model mice glomerular [43]. Carla Iacobini found that kidney mRNA levels of fibronectin, laminin, collagen IV, and TGF-β were up-regulated in mice with using intraperitoneal injection of AGEs for three months [44]. Our results found that renal function was injured in AGEs treated WT group compared with control WT group, the expression and activity of SphK1 and S1P formation also elevated in AGEs treated WT group. BUN, 24 h UP levels, the expression of FN and kidney injury was obviously ameliorated in AGEs treated SphK1^-/-^ group compared with AGEs treated WT group. It was indicated that AGEs could elevate the expression and activity of SphK1, SphK1 knockout could reverse AGEs-induced kidney injury in mice.

Although we have proved that SphK1 plays an important role in AGEs-induced diabetic kidney fibrosis, however, it is unclear how AGEs regulate the expression and activity of SphK1. There are many lysine residues which could be post-translational modification on the SphK1 protein. Shimin Zhao used LC/LC-MS/MS to analyze protein lysine acetylation, they found that many metabolic enzyme could be acetylation include SphK1 [35]. Hongyang Yu found that acetylation of SphK1 increases protein stability through inhibiting its ubiquitination [34]. AGEs could decrease Sirt1 levels in terms of protein expression and activity in GMCs, on the other hand, AGEs could activate the activity of p300 [45, 46]. It was provided theory for AGEs regulate SphK1 degradation by affecting the SphK1 post-translational modification. Our study found that SphK1 can be ubiquitinated and further be polyubiquitinated include K48-linked and K63-linked ubiquitin chains modified. We also found that AGEs could prolong SphK1 half-life compared with control. It was preliminary indicated that AGEs could possibly regulate SphK1 protein by affecting its degradation. The mechanism of AGEs regulating SphK1 is needed to be further investigated.

## MATERIALS AND METHODS

### Reagents and antibodies

D-glucose was purchased from Amresco (Solon, OH, USA). Bovine serum albumin (BSA, Fraction V) was obtained from Mbchem (Shanghai, China). SphK1 inhibitor 5C (5C) was obtained from Cayman Chemical Company (Ann Arbor, Michigan, USA). MG-132 and SphK1 inhibitor PF-543 were obtained from Selleck. RAGE inhibitor FPS-ZM1 and RAP were obtained from Merck Millipore. C17-D-erythro-Sphingosine 1-Phosphate (C17-S1P), S1P, and C17-D-erythro-sphingosine (C17-sph) were purchased from Avanti Polar Lipids (Alabaster, AL, USA). Streptozotocin (STZ), aminoguanidine (AG), ATP, cycloheximide (CHX) and antibodies against α-tubulin were purchased from Sigma-Aldrich Corporation (St. Louis, MO, USA). Polyvinylidene difluoride (PVDF) membranes were purchased from Immobilon^®^-PSQ (Millipore, MA, USA). Nuclear Extract Kit was purchased from Active Motif (Carlsbad, CA, USA). Antibodies against FN, RAGE, OctA(FLAG^®^), SphK1 used for IP (Santa Cruz Biotechnology, Santa Cruz, CA, USA); SphK1 used for WB (Cell Signaling Technology, Boston, MA, USA); SphK1 used for immunohistochemistry (Boster Immunoleader, Wuhan, Hubei, China); horseradish peroxidase-conjugated secondary antibodies (Promega, Madison, WI, USA); Lipofectamine® LTX & Plus Reagent and Lipofectamine® RNAiMAX Reagent (Life Technologies™, Grand Island, NY, USA) were purchased from commercial sources.

### Preparation and characterization of AGEs

AGEs-BSA (AGEs) was produced using D-glucose and fatty acid free BSA as described previously [17]. Briefly, 0.8 g of BSA (40 mg/mL) was incubated with 1.8 g of D -glucose in 20 mL of PBS (0.2 M, pH 7.4) under sterile conditions at 37 °C for 90 days without light. All preparations of AGEs were dialyzed in 10 mM of PBS (pH 7.4) for 96 h to remove the free glucose and passed over detoxigel columns (Detoxi-Gel™ Endo-toxin Gel; Thermo Fisher Scientific, Rockford, IL, USA) to remove endotoxin. Endotoxin levels in preparations were further determined via limulus amebocyte lysate testing (Houshiji, Xiamen, China), and were found to be less than 0.01 EU/mL. Estimation of glycation by spectrofluorometry (PerkinElmer, Waltham, MA, USA) with excitation wavelength of 370 nm and emission wavelength of 440 nm revealed approximately 50-fold increase in characteristic fluorescence for AGEs as compared with control.

### Cell culture

Rat primary GMCs were obtained from Sprague Dawley rat kidney cortex fragments using standard protocols as described previously [27].

### Plasmids and transient transfection

The vector (pcDNA3 plasmid), Flag-tagged human wild-type SphK1 (SphK^WT^) and the dominant-negative SphK1 (SphK^G82D^) were kindly provided by Dr. Pu Xia (Australia) [36]. Vector pEGFP, human full-length wild-type pEGFP-RAGE and human cytoplasmic-deleted mutant-type pEGFP-RAGEΔcyto were constructed and supplied by Genechem. PcDNA3-HA-Ub, pcDNA3-HA-Ub K0, vector pRK5, pRK5-HA-Ub, pRK5-HA-Ub K48, and pRK5-HA-Ub K63 were purchased from Addgene (http://www.addgene.org/). GMCs were plated in 35 mm culture plates 24 h prior to transfection. Then, the cells were transfected with 2 μg indicated plasmid using Lipofectamine^®^ LTX & Plus Reagent. GMCs were incubated for 72 h to harvest, and western blot assay or SphK activity detection was performed.

### Small interfering RNAs (siRNAs) and silencing experiment

Validated stealth negative control, SphK1-specific siRNA oligonucleotides and RAGE-specific siRNA oligonucleotides were obtained from Life Technologies. The special sequences of SphK1-siRNA were Sense:5’-GCAGCUUCCUUGAACCAUUTT-3’, Antisense:5’-AAUGGUUCAAGGAAGCUGCTT-3’. The special sequences of RAGE-siRNA: sense, 5’-GGAAACCUCUGAUUCCUGAUGGCAA-3’ and antisense, 5’-UUGCCAUCAGGAAUCAGAGGUUUCC-3’. GMCs were transfected with the indicated siRNA oligonucleotides using lipofectamine RNAiMAX reagent according to the instructions of the manufacturer.

### Western blot assay

Western blot assay was performed as previously described [27].

### SphK activity assay

SphK activity was determined as our previous published paper [47]. The reaction was initiated by adding 2.5 mL of 200 mM C17-Sph (dissolved in 5% Triton X-100) and 2.5 mL of 20 mM ATP containing MgCl_2_ (200 mM) to a final volume of 50 mL. After incubation at 37 °C for 20 min, the reaction was terminated with 5 mL of 1 M HCl followed by 200 mL of chloroform: methanol: HCl solution (100: 200: 1, v/v). S1P (10 ng) was added as an internal standard. After vigorous vortexing, 60 mL of chloroform and 60 mL of 2 M KCl were added. The phases were separated by centrifugation at 12,000 g for 5 min at 4 °C. The lower chloroform phase was transferred into a 1.5 mL Eppendorf tube and then vacuum-dried in a SpeedVac for 60 min at room temperature. The dried residue was reconstituted in 100 mL mobile phase (methanol: 0.1% formic acid = 95:5, v/v) and then vortex-mixed vigorously for 1 min. A 10 mL aliquot of the final solution was injected directly into a liquid chromatography tandem-mass spectrometry (LC-MS/MS) system (Thermo Finnigan, Silicon Valley, CA, USA) for analysis.

### S1P quantification assay

S1P was quantified using the LC-MS/MS assay following the method as previously described [48]. 1 ×10^6^ GMCs were washed twice with ice-cold PBS, harvested by scraping with blade in 500 mL of ice-cold PBS and transferred to 4 mL prechilled eppendorf tube on ice. 10 mg of kidney tissues were homogenized in 500 mL of ice-cold PBS using a tissue homogenizer. Then 10 mL of internal standard (10 ng/mL for C17-S1P), 500 mL of methanol and 100 mL of 6 M HCl were added into the cell pellets or kidney tissues homogenates. After vortex-mixed vigorously for 10 s, 1 mL of chloroform was added and vortex-mixed vigorously for at least 2 min. For fast segregation of the organic and aqueous phases, the samples were centrifuged for 3 min at 1900 g. The lower organic phase was transferred to a new centrifuge tube. A second chloroform extraction (1 mL) was performed and the two organic phases were combined. After evaporating chloroform in a vacuum concentrator for 60 min at 50 °C, the dried residue was reconstituted in 100 mL of mobile phase (methanol: 0.1% formic acid = 95:5, v/v). Subsequently, the mixture was vortex-mixed for 1 min followed by centrifugation at 14,000 g for 5 min at 4 °C. The supernatant was transferred into clean glass vials. A 10 mL aliquot of the supernatant was injected into the LC-MS/MS system for analysis.

### Coimmunoprecipitation experiment

Coimmunoprecipitation experiment was determined as our previous published paper [45].

### STZ-induced diabetic mice model and grouping mice

All animal procedures conformed to the China Animal Welfare Legislation and were reviewed and approved by the Sun Yat-sen University Committee on Ethics in the Care and Use of Laboratory Animals. Male C57 mice (bodyweight: 20 ± 2g) from Laboratory Animal Center, Sun Yat-sen University, Guangzhou, China. After fed with regular diet for one week, mice were fasted for 8 h and then given intraperitoneal injections of 50 mg/kg streptozotocin (STZ) or the vehicle daily for 5 consecutive days as previously reported [49, 50]. After 72 h completion of STZ injections, the C57 mice blood samples were collected via the tail vain, and fasting blood glucose (FBG) levels were measured using an onetouch ultra blood glucose meter (Johnson & Johnson Co., New Brunswick, NJ, USA). Mice with FBG levels above 11.1 mM were considered as diabetic. Diabetic mice were randomized (8/group) to receive aminoguanidine (100 mg/kg, intragastric administration, daily) or orally given equal volumes of physiological saline. Control mice (8/group) were also fed equal volumes of physiological saline. Treatment was continued for 16 weeks, at which time mice were sacrificed.

### STZ-induced SphK1^-/-^ diabetic mice model and grouping mice

Male wild type (WT) mice (bodyweight: 20 ± 2g) from Laboratory Animal Center, Sun Yat-sen University, Guangzhou, China. Male SphK1^-/-^ mice (bodyweight: 20 ± 2g) were kindly provided by Richard L. Proia, NIH [51]. After created diabetic model by STZ successfully, WT diabetic mice (8/group), WT control mice (8/group), SphK1^-/-^ diabetic mice (8/group), SphK1^-/-^ control mice (8/group) were continually given free access to water and standard laboratory chow. Animal experiments were continued for 16 weeks, at which time mice were sacrificed.

### AGEs-induced SphK1^-/-^ diabetic mice model and grouping mice

Male WT mice (bodyweight: 20 ± 2g) from Laboratory Animal Center, Sun Yat-sen University, Guangzhou, China. Male SphK1-/- mice (bodyweight: 20 ± 2g) were kindly provided by Richard L. Proia, NIH. Mice were given intraperitoneal injections of 200 mg/kg AGEs (8/group) or BSA (8/group) daily for 6 consecutive days per week. Animal experiments were continued for 12 weeks, at which time mice were sacrificed.

### Biochemical analysis, morphological study and Immunohistochemistry

FBG levels were determined and urine was collected from the mice housed in metabolic cages for 24 h. All animals were sacrificed after anesthesia, blood samples were collected. Kidneys samples were quickly excised, weighed, and fixed in 10% neutral buffered formalin, or frozen in liquid nitrogen and then stored at -80 °C.

FBG levels were measured using a one touch ultra blood glucose meter. Blood urea nitrogen (BUN), serum creatinine (Cr), and 24h urine protein (UP) were analyzed by the Department of Pathology, the First Affiliated Hospital, Sun Yat-sen University. Renal hypertrophy was assessed using the kidney weight to body weight ratio (KW/BW).

Kidney samples fixed in 10% neutral buffered formalin were then embedded in paraffin; sections of kidney (2 μm of thickness) were prepared and stained with periodic acid-schiff (PAS) and hematoxylin & eosin (HE) by the Department of Pathology, the First Affiliated Hospital, Guangzhou Medical University. Glomerular lesions were scored semiquantitatively by a blinded observer who examined 30 randomly selected glomeruli in each specimen using glomerular sclerosis score [52]. Immunohistochemical staining sections of kidney were processed using a standard immunostain protocol as previously reported [53].

### Statistical analysis

Values were expressed as means ± SD. All data were assessed by the Graphpad Prism 5.0 software. Unpaired student’s t test was used for comparison between two groups. For multiple comparisons, data were analyzed by one-way ANOVA with post hoc multiple comparisons. Independent experiments were performed at least thrice with similar results. *P* < 0.05 was considered statistically significant.

## CONCLUSION

In summary, our study found that AGEs-RAGE could upregulate FN through regulating SphK1 in cell experiments and we also verify those results *in vivo* using STZ-induced SphK1^-/-^ mice as well as AGEs-induced mice. Our study confirmed that SphK1 plays important roles in AGEs-induced DN, and AGEs possibly regulate SphK1 stability and protein expression by decreasing its ubiquitination. Together, inhibition SphK1 signaling in the kidney may be a promising treatment strategy in DN.

## Abbreviations

AG: aminoguanidine
AGEs: advanced glycation-end products
AP-1: activator protein 1
BUN: blood urea nitrogen
C17-S1P: C17-D-erythro-sphingosine 1-phosphate
C17-sph: C17-D-erythro-sphingosine
CHX: cycloheximide
Cr: serum creatinine
DMEM: Dulbecco’s modified Eagle’s medium
DN: diabetic nephropathy
ECM: extracellular matrix
FBG: fasting blood glucose
FN: fibronectin
GBM: glomerular basement membrane
GMCs: glomerular mesangial cells
HG: high glucose
KW/BW: kidney weight to body weight ratio
LC-MS/MS: liquid chromatography tandem-mass spectrometry
MMI: mesangial matrix index
NG: normal glucose
RAGE: receptor for AGEs
PAS: periodic acid-schiff
RBG: random blood glucose
ROS: reactive oxygen species
S1P: sphingosine 1-phosphate
siRNA: small interfering RNA
SphK: sphingosine kinase
STZ: streptozotocin
TGF-β1: transforming growth factor-beta 1
UP: urine protein
5C: SphK1 inhibitor 5C
